# Deciphering Myostatin’s Regulatory, Metabolic, and Developmental Influence in Skeletal Diseases

**DOI:** 10.3389/fgene.2021.662908

**Published:** 2021-03-29

**Authors:** Catherine L. Omosule, Charlotte L. Phillips

**Affiliations:** ^1^Department of Biochemistry, University of Missouri, Columbia, MO, United States; ^2^Department of Child Health, University of Missouri, Columbia, MO, United States

**Keywords:** myostatin, osteoblast, osteoclast, osteocyte, osteogenesis imperfecta, osteoporosis, Duchenne muscular dystrophy, diabetes

## Abstract

Current research findings in humans and other mammalian and non-mammalian species support the potent regulatory role of myostatin in the morphology and function of muscle as well as cellular differentiation and metabolism, with real-life implications in agricultural meat production and human disease. Myostatin null mice (*mstn^−/−^*) exhibit skeletal muscle fiber hyperplasia and hypertrophy whereas myostatin deficiency in larger mammals like sheep and pigs engender muscle fiber hyperplasia. Myostatin’s impact extends beyond muscles, with alterations in myostatin present in the pathophysiology of myocardial infarctions, inflammation, insulin resistance, diabetes, aging, cancer cachexia, and musculoskeletal disease. In this review, we explore myostatin’s role in skeletal integrity and bone cell biology either due to direct biochemical signaling or indirect mechanisms of mechanotransduction. *In vitro*, myostatin inhibits osteoblast differentiation and stimulates osteoclast activity in a dose-dependent manner. Mice deficient in myostatin also have decreased osteoclast numbers, increased cortical thickness, cortical tissue mineral density in the tibia, and increased vertebral bone mineral density. Further, we explore the implications of these biochemical and biomechanical influences of myostatin signaling in the pathophysiology of human disorders that involve musculoskeletal degeneration. The pharmacological inhibition of myostatin directly or *via* decoy receptors has revealed improvements in muscle and bone properties in mouse models of osteogenesis imperfecta, osteoporosis, osteoarthritis, Duchenne muscular dystrophy, and diabetes. However, recent disappointing clinical trial outcomes of induced myostatin inhibition in diseases with significant neuromuscular wasting and atrophy reiterate complexity and further need for exploration of the translational application of myostatin inhibition in humans.

## Introduction

Contemporary research support the potent regulatory effects of myostatin in living organisms including humans, other mammalian (cattle, pigs, monkeys, mice, rats), and non-mammalian species (frogs, common carp) (Mendias et al., 2008; Zhong et al., 2016). Spurred by the début of the “mighty mice” by McPherron et al. (1997), the study of myostatin has unveiled a cadre of influences in muscle morphology and function and likewise cellular differentiation and metabolism, with real-life implications in agricultural meat production and human diseases (McPherron et al., 1997). Myostatin is expressed in the myotome compartment of developing somites during embryogenesis (McPherron et al., 1997). Myostatin further influences primitive mesenchymal stem cell (MSC) progenitor lineage differentiation, resulting in widespread skeletal muscle hyperplasia and hypertrophy as observed in myostatin null (*mstn^−/−^*) mice (Cho et al., 2002). Nonetheless, myostatin’s reach extends beyond the musculoskeletal system. Regulatory roles of myostatin have now been discovered in aging, cancer cachexia, insulin sensitivity, diabetes, and in cardiac tissue where myostatin influences inflammation (McPherron and Lee, 2002; Yarasheski et al., 2002; Rodgers and Garikipati, 2008; Guo et al., 2009; Zhang et al., 2011; Biesemann et al., 2014, 2015; Jackson et al., 2014; Singh et al., 2014; Loumaye et al., 2015; Dong et al., 2016; Kong et al., 2018).

**Figure 1:**
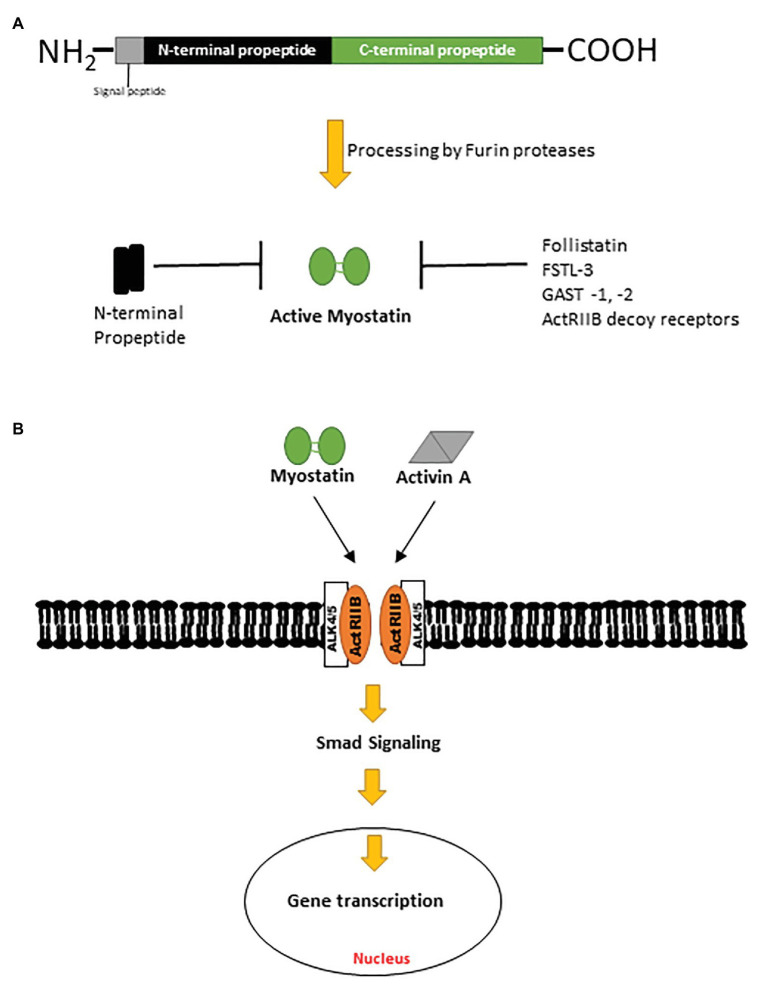
**(A)** Pre-promyostatin is synthesized as a 375 amino acid peptide with a signal peptide domain and N‐ and C-terminal domains. Cleavage by furin proteases generate an active myostatin C-terminal dimer which can then form non-covalent complexes with the N-terminal fragments, becoming latent. **(B)** Active myostatin and activin A effect canonical cellular changes *via* the activin receptor type II B and intracellular Smad signaling cascades.

Myostatin, also known as growth differentiation factor 8 (GDF8), is a negative regulator of muscle mass and a member of the TGF-β superfamily of proteins (McPherron et al., 1997). Conserved across species, myostatin shares 89% sequence homology with growth differentiation factor 11 (GDF11), another TGF-β ligand (McPherron et al., 1997; Gad and Tam, 1999; Poggioli et al., 2016). Myostatin is initially synthesized by myocytes as a pre-promyostatin molecule composed of an N-terminal signal sequence (for secretion), an N-prodomain region (essential for proper folding of myostatin and subsequently proteolytically processed), and the biologically active C-terminal domain. The precursor pre-promyostatin must undergo proteolytic cleavage to form the biologically active myostatin molecule, which exists as a disulfide-linked dimer of two C-terminal domains (Lee and McPherron, 2001; Favia et al., 2019; Figures 1A,B). The cleaved propeptide domain also plays a regulatory role through non-covalent binding to the active myostatin C-terminal domain to form an inactive latent myostatin complex (Bogdanovich et al., 2005). Other natural inhibitors of myostatin include follistatin, follistatin-like 3 (FSTL-3), and GDF-associated serum protein 1 and 2 (GASP-1 and -2; Figure 1A; Laurent et al., 2016). In its active form, myostatin binds and signals primarily through activin receptor type II B (ActRIIB), a serine/threonine kinase, which dimerizes with activin receptor-like kinase 4 (ALK4) and effects changes in the Smad signaling pathway (Figure 1B). Myostatin is also capable of effecting a non-canonical signaling cascade involving the cellular energy-sensing enzyme AMP-activated kinase (AMPK) and a regulatory protein kinase transforming growth factor-β-activated kinase 1 (TAK1; Biesemann et al., 2014; Rybalka et al., 2020).

Agricultural benefits of myostatin inhibition include increasing feed efficiency and lean muscle mass in farm animals which improves meat production. For example, Meishan pigs, known to have poor feed efficiency and high carcass fat, exhibit a double-muscled phenotype concomitant with hyperplastic muscle fibers and decreased body fat in the absence of myostatin (Qian et al., 2015). Interestingly, 20% of mutant pigs also had an extra thoracic vertebrae, which implicates myostatin in the formation and patterning of thoracic vertebrae in pigs (Qian et al., 2015). Further, two species of cattle with known myostatin deficiencies, the Belgian Blue and Piedmontese cattle, have muscle hypertrophy and higher lean muscle to bone mass ratios relative to normal cattle (Shahin and Berg, 1985; Grobet et al., 1997). The muscle hypertrophy present in pigs and cattle deficient in myostatin is due to muscle fiber hyperplasia in contrast to the muscle hypertrophy seen in myostatin knock-out mice where both hypertrophic and hyperplastic muscle fibers are observed (McPherron et al., 1997; McPherron and Lee, 1997).

In humans, skeletal muscle mass and function significantly decline after only 5 days of disuse, and is accompanied by increased expression of myostatin (Dirks et al., 2014; Wall et al., 2014). A study by Gonzalez-Cadavid et al. (1998) which characterized the expression of myostatin in human skeletal muscle and serum, found an inverse correlation between serum myostatin levels and fat-free mass. Further, human immunodeficiency virus (HIV)-infected men had elevated levels of serum and intramuscular myostatin relative to healthy men. HIV-infected men who had lost in excess of 10% of their premorbid weight and were considered to have acquired immunodeficiency syndrome (AIDS) also had higher serum myostatin levels than HIV-infected men without AIDS, suggesting that increased intramuscular myostatin production contributes to the AIDS-associated muscle wasting syndrome (Gonzalez-Cadavid et al., 1998).

The pharmacological inhibition of myostatin using myostatin-specific antibodies or ligand traps that inhibit ActRIIB binding and/or its signaling have demonstrated significant increases in muscle mass in multiple mouse models of aging and disease (Arounleut et al., 2013; DiGirolamo et al., 2015; Latres et al., 2017; Jeong et al., 2018a; Tauer and Rauch, 2019). While some of these welcomed increases may boost muscle function (Jeong et al., 2018a) and bone integrity (Chen et al., 2017; Jeong et al., 2018b), genetic myostatin deficiency in the golden retriever muscular dystrophy (GRippet) dog resulted in unequal occurrences of hypertrophy and atrophy in different muscle groups (Kornegay et al., 2016). Muscle imbalance was reflected by hypertrophy in the cranial sartorius flexor muscles and atrophy of the quadriceps femoris extensor muscles. Worsening of joint contractures, physical decline, postural instability, and heightened disease severity in GRippet dogs was consequently observed (Kornegay et al., 2016). Moreover, although muscle in *mstn^−/−^* mice are larger, they have compromised force production (Amthor et al., 2007; Gentry et al., 2011). This suggests that while impressive changes in musculature may be observed in smaller animal models, additional studies remain to be done to validate myostatin’s role and potential in ameliorating muscle-related human disorders.

It is noteworthy that most of the data available on myostatin’s role in bone homeostasis is based on mouse studies. In humans and monkeys, serum myostatin levels are ~5 and 9 times less than that found in mice respectively, whereas serum levels of activin A, another ligand in the TGF-β superfamily are greater in monkeys and humans (Latres et al., 2017). Recent studies evaluating anti-myostatin and anti-activin A antibodies in primates suggests myostatin alone is a less potent contributor to muscle mass regulation in primates (Schuelke et al., 2004; Latres et al., 2017). Thus, to obtain maximal increases in muscle mass in primates, a combinatory myostatin and activin A antibody neutralization treatment was required even though no changes in femoral bone mineral density (BMD) or mineral content (BMC) were observed (Latres et al., 2017). Combinatorial treatment approaches in humans thus may be more efficacious. Studies on muscle fiber number and size in myostatin null humans are currently unavailable although the effect of myostatin deficiency on muscle fiber number and size appear to be species-specific (McPherron et al., 1997; McPherron and Lee, 1997).

The ubiquitous nature of skeletal muscle has fueled extensive investigations of myostatin’s role in skeletal muscle energy and metabolic homeostasis. However, there is a paucity in our understanding of myostatin’s role in bone homeostasis. As a mechanosensitive tissue, the skeleton accommodates increasing muscle mass by adjusting its strength and mass so as not to fracture (Frost, 2003). This review summarizes our current understanding of the influence of myostatin on bone cell metabolism and differentiation (direct regulator) and whole bone phenotypes (indirect regulator; *via* mechanosensation) of animal models partially or completely devoid of myostatin and highlights investigations of the skeletal impact of genetic and pharmacological inhibition of myostatin in osteogenesis imperfecta (OI), osteoporosis, osteoarthritis, Duchenne muscular dystrophy (DMD) and diabetes; pulling together data from human, murine, and canine studies.

## Myostatin in Bone

Newborn myostatin null mice (*mstn^−/−^*) possess larger vertebral bone volume fraction (bone volume/total volume; BV/TV), due to increased BV, trabecular thickness (Tb.Th), and bone area (BA), as well as increased tissue mineral density (TMD) and BMD as compared to newborn wildtype (Wt) mice (Suh et al., 2020). Ossification in the cranium and sternum is also significantly enhanced (Suh et al., 2020). The superior bone microarchitectural properties observed at birth in *mstn^−/−^* mice were maintained at 10-week of age, as reflected by higher BV, BMD and BMC compared to aged-matched Wt mice. In addition, BV, cortical thickness and TMD in tibia and vertebral BMD were elevated in the 10 weeks old *mstn^−/−^* mice (Suh et al., 2020). Primary *mstn^−/−^* chondrocytes also express significant amounts of *Col10a1* mRNA, a marker of hypertrophic chondrocytes further supporting a role for myostatin in the regulation of bone development (Suh et al., 2020).

Studies by Elkasrawy and Hamrick (2010) and Elkasrawy et al. (2012)have shown enhanced skeletal phenotypes in *mstn^−/−^* and myostatin-deficient (*mstn^+/−^*) mice. These increases were observed at regions of muscle insertion and linked to bone’s biomechanical interactions with the increased muscle masses (mechanosensation). For example, triceps and deltoid muscles at the deltoid crest (point of insertion) in *mstn^−/−^* mice were significantly larger than in Wt mice. At the points of insertion of these muscles onto the humerus, higher cortical BA, BMC, and periosteal circumference were also observed (Elkasrawy and Hamrick, 2010).

Osteoblasts (bone-forming cells) work in concert with osteoclasts (bone-resorbing cells) and osteocytes (mechanosensing cells) to maintain skeletal homeostasis. In the following section, *in vitro* studies characterizing the response of these bone cell types to endogenous or exogenous myostatin deficiency are discussed to provide further evidence of the cellular and molecular mechanistic impact of myostatin in skeletal metabolism (Table 1).

**Table 1:** The effect of myostatin inhibition on bone cells.

Type of cell	Source of myostatin	Cellular outcome
Osteoblasts	Endogenous (*mstn^−/−^*)	Increased expression of osteogenic markers (Suh et al., 2020)
Bone marrow-derived mesenchymal stem cells (BMSCs)	Endogenous (*mstn^−/−^*)	Increased differentiation potential (Hamrick et al., 2007)
Osteoblasts	Exogenous	Dose-dependent decreases in differentiation and mineralization (Chen et al., 2017, p. 8)
Human fetal osteoblasts	Exogenous	Decreased proliferation (Wu et al., 2018)
Bone marrow macrophages (BMMs)	Endogenous (*mstn^−/−^*)	Decreased proliferation (Dankbar et al., 2015)
Osteoclasts	Endogenous (*mstn^−/−^*)	Increased osteoclastogenesis (Dankbar et al., 2015)
Osteoclasts	Exogenous	Increased number of and larger TRAP+ multinucleated osteoclasts (Dankbar et al., 2015; Chen et al., 2017)
Osteocytes	Exogenous	Decreased expression of miR-218 (Qin et al., 2017)

### Osteoblasts

Osteoblasts, fibroblasts, bone-marrow-derived stromal cells, chondrocytes, and bone-marrow-derived MSCs (BMSCs) express the membrane receptor ActRIIB (Hamrick et al., 2007; Elkasrawy et al., 2012). Primary murine calvarial osteoblasts exhibit a dose-dependent response to myostatin as evidenced by decreasing differentiation and mineralization with increasing myostatin (Chen et al., 2017). Alkaline phosphatase (*Alpl*) activity and osteocalcin (*Bglap*) secretion are also decreased, as is the expression of the osteoblast transcription factors, runt-related transcription factor 2 (*Runx2*), and osterix (*Osx*; Chen et al., 2017).

In an elegant phenotypic characterization study, Suh and colleagues showed that primary *mstn^−/−^* osteoblasts have higher differentiation potential demonstrated by the upregulated expression of the osteogenic markers; *Runx2*, transcription factor 7 (*Sp7*), *Alpl*, *Bglap*, bone sialoprotein 2 (*Ibsp*) and osteopontin precursor (*Spp1*) relative to Wt primary osteoblasts (Suh et al., 2020). Also, bone morphogenetic protein 2 (*Bmp2*), *Smad-1* and *-9*, and insulin growth factor 1 (*IGF-1*) expression were elevated (Hamrick et al., 2007; Suh et al., 2020). BMSCs from *mstn^−/−^* mice also had higher differentiation potential vs. Wt BMSCs. While mechanical stimuli increased the expression of *Runx2*, *IGF-1*, *Bmp2*, and periostin (*Osf-2*), addition of exogenous recombinant myostatin did not change the osteogenic potential of *mstn^−/−^* BMSCs *in vitro* (Hamrick et al., 2007). Pre-treatment with recombinant myostatin, however, diminished the expression of osteogenic markers in *mstn^−/−^* BMSCs when mechanically stimulated (Hamrick et al., 2007). Further, exogenous myostatin inhibited proliferation in human fetal osteoblasts in a dose-dependent manner as reflected by decreased ALPL, BGLAP, and RUNX2 mRNA levels (Wu et al., 2018).

Lastly, Wt C57BL6/J male and female mice exhibited increased femoral osteoblast number when treated with a soluble activin receptor type IIB decoy molecule (sActRIIB-mFc) designed as a ligand trap for circulating myostatin (Jeong et al., 2018b). Female sActRIIB-mFc-treated Wt mice also exhibited increased periosteal mineralizing surface (MS/BS), mineral apposition rate (MAR), and bone formation rate (Jeong et al., 2018b).

### Osteoclasts

Both precursor and mature osteoclasts, but not macrophages, express myostatin (Dankbar et al., 2015). In osteoclasts, myostatin simultaneously activates ERK-dependent osteoclastogenic target genes independent of ERK while stimulating *pSMAD*2-dependent nuclear translocation of nuclear factor of activated T-cells (NFATC1; Dankbar et al., 2015). This culminates in increased expression of the key osteoclastogenic genes for integrin αv, integrin β3, DC-STAMP, and the calcitonin receptor (Dankbar et al., 2015).

Primary *mstn^−/−^* osteoclasts have decreased expression of the osteoclastogenic markers: *Nfatc1*, *Fos*, *Src*, tartrate-resistant acid phosphatase type 5 precursor (*Acp5*) and Cathepsin K (*Ctsk*; Suh et al., 2020). Treatment of C57BL/6 bone marrow monocytes and macrophages with recombinant myostatin increased tartrate-resistant acid phosphatase (TRAP)-positive multi-nucleated osteoclasts, enhanced TRAP activity, and increased *Nfatc1* expression in a dose-dependent manner (Chen et al., 2017). To further explore the potential of myostatin to directly regulate osteoclastogenesis, Dankbar et al. (2015) differentiated bone marrow macrophages (BMMs) into osteoclasts in the presence of myostatin using receptor activator of nuclear factor κB ligand (RANKL) and macrophage colony stimulating factor (M-CSF), two critical ligands in osteoclast formation (Park et al., 2017). Resultant osteoclasts were larger with enlarged cytoplasmic compartments (x8) and more nuclei (x7), implying that myostatin considerably augments RANKL-induced osteoclastogenesis (Dankbar et al., 2015). In addition, BMMs from *mstn^−/−^* mice were smaller, less proliferative (−44%), and had half the number of nuclei as BMMs from Wt mice. Although *mstn^−/−^* osteoclasts generated a larger number of resorption pits *in vitro*, no differences in resorption area per pit were observed. Thus, myostatin stimulates osteoclastogenesis, but does not appear to impact osteoclast activity (Dankbar et al., 2015).

Further, to investigate the effect of ligands secreted from *mstn^−/−^* osteoblasts on osteoclastogenesis, *mstn^−/−^* osteoblasts were co-cultured with Wt BMMs (Dankbar et al., 2015). This resulted in the formation of 90% less osteoclasts compared to a co-culture of Wt osteoblasts with Wt BMMs. Likewise, *mstn^−/−^* BMMs co-cultured with Wt osteoblasts resulted in 50% less osteoclasts. Osteoclastogenesis and likewise skeletal metabolism are thus likely regulated by myostatin (Dankbar et al., 2015).

Lastly, histological analyses of Wt C57BL6/J mice treated for 11 weeks with sActRIIB-mFc, revealed decreased osteoclast activity in female mice only (Jeong et al., 2018b). Additional histological characterization of *mstn^−/−^* tibias revealed a 34% decrease in osteoclast number (Dankbar et al., 2015).

### Osteocytes and Downstream Effects

Osteocytes, considered as both mechanosensors and mechanotransducers, constitute about 95% of bone cells (Bonewald, 2011). Osteocytes relay mechanical signals from normal loading or its absence by secreting factors that influence osteoblast and osteoclast function (Bonewald, 2011). Myostatin can directly modulate key transcriptional and osteogenic factors at both the transcriptional and translational levels (Qin et al., 2017). Treatment of the osteocytic cell-line Ocy454 with recombinant myostatin upregulated the mRNA and protein levels of RANKL, and sclerostin (*Sost*) and dickkopf-1 (*Dkk1*; Qin et al., 2017) inhibiting the WNT/β-catenin signaling pathway, which is activated in response to bone mechanical loading, facilitating the transduction of mechanical forces by osteocytes into chemical signals (Bonewald and Johnson, 2008).

MicroRNAs are small single-stranded non-coding RNAs with regulatory roles in biological pathways, like bone cell differentiation and function (Qin et al., 2017). MicroRNA-218 (miR-218) regulates the WNT pathway and inactivates *Sost* and *Dkk2* during osteoblast differentiation (Maeda et al., 2012). Osteocytes treated with myostatin expressed less miR-218; and released osteocytic exosomes (30–100 nm microvesicles) which also had less miR-218, consistent with increased expression of *Sost* and *Dkk2* (Qin et al., 2017). MC3T3 preosteoblastic cells administered the resulting myostatin-modified osteocytic exosomes were less likely to differentiate and had lower levels of the activated β-catenin protein (Qin et al., 2017). In contrast, myostatin-modified osteocytic exosomes did not impact osteoclast proliferation and differentiation (Qin et al., 2017). These studies, therefore, suggest that myostatin impacts bone metabolism by regulating osteocytic control over osteoblast differentiation.

## Implications For Human Diseases

Myostatin has been an attractive target for treating musculoskeletal dysplasias associated with inferior muscle and bone function as seen in osteoporosis, OI, and DMD since myostatin regulates muscle metabolism and homeostasis. In 2004, a loss of function mutation in the myostatin gene was reported in an infant (Schuelke et al., 2004). In addition to the significant reduction in his subcutaneous fat pad (decreased adiposity), the cross-sectional area of his quadriceps was 7.2 SD above the mean of his age-matched cohort. Superior muscle strength and mass was observed when he was about 4 years old, shown in his ability to horizontally suspend two 3 kg dumbbells (Schuelke et al., 2004). At the time of this writing, no additional information regarding the patient’s bone health, or the effect of genetic myostatin mutations in human bone has been reported.

Homozygous myostatin null mice (*mstn^−/−^*) have global increases in skeletal muscle mass and are about 30% larger than their Wt littermates (McPherron et al., 1997). Inducing myostatin knock-out in the skeletal muscle of adult mice also increases muscle mass, suggesting that post-developmental changes in skeletal mass accrual can occur (Welle et al., 2007). In mice, increased femoral BMD is associated with the absence of myostatin (Hamrick, 2003). Also, myostatin directly regulates bone cell metabolism and differentiation and is expressed during fracture repair, acting as a negative regulator of callus size, and potentially influencing fracture healing (Kellum et al., 2009). Myostatin expression was maximal a day after fracture in tibia calluses suggesting a regulatory role in primitive MSC recruitment and differentiation during the inflammatory stage of healing (Cho et al., 2002). Also, a fibular osteotomy model in *mstn^−/−^* and *mstn^−/+^* mice revealed larger and biomechanically stronger fracture calluses 4 weeks after fracture, relative to Wt mice (Kellum et al., 2009). Three treatments of 20 mg/kg recombinant GDF8 propeptide [inactive N-terminal region of myostatin; capable of inhibiting active myostatin (Bogdanovich et al., 2005)] in 4–6 months old mice with fibula osteotomy also increased callus volume (Hamrick et al., 2010). Thus, myostatin likely regulates fracture healing and callus formation by inhibiting the recruitment and proliferation of progenitor cells (Kellum et al., 2009).

### Osteogenesis Imperfecta

Osteogenesis Imperfecta is a heritable genetic connective disorder that arises primarily from alterations in type I collagen structure or processing and results in fragile bones that break with minimal trauma. Classical autosomal dominant OI is grouped into four types according to disease severity: I (mild OI); II (perinatally lethal OI); III (most severe survivable form of OI); and IV (moderately severe OI; Sillence et al., 1979). Functional muscle deficits have been identified in children with mild to moderate OI even when their relatively smaller muscle masses have been taken into account (Veilleux et al., 2014, 2017). Thus, treatments that improve both muscle and bone properties in OI regardless of the underlying mutation or disease severity promise to be beneficial. Several mouse models representing different mutations and OI disease severities have been challenged with genetic or pharmacological inhibition of myostatin alone or in combination with other TGF-β ligands including activin A (DiGirolamo et al., 2015; Oestreich et al., 2016a,b; Jeong et al., 2018a,b; Tauer and Rauch, 2019).

The *Col1a2^oim^* mouse arose from a spontaneous mutation in *Col1a2* resulting in a non-functional protein (Chipman et al., 1993). Heterozygote *oim* (*+/oim*) mice model mild human OI whereas homozygote *oim* (*oim/oim*) model a severe phenotype (Saban et al., 1996). In a recent study by Oestreich and colleagues, Wt and *+/oim* offspring born to myostatin deficient dams exhibit improved skeletal phenotypes in adulthood compared to those born to Wt and *+/oim* dams (Oestreich et al., 2016b). To investigate whether intrinsic biological processes pre‐ or post-implantation were driving the changes, *+/oim* embryos were transferred to *+/mstn* dams and *+/oim* dams (control) at d3.5 gestational age. The offspring born to recipient *+/mstn* dams also had increased femoral strength in adulthood as compared to offspring born to recipient *+/oim* dams suggesting that reduced maternal myostatin levels could confer skeletal advantages to offspring post-conception *via* alterations in prenatal developmental programming (Oestreich et al., 2016b). In another study, congenital myostatin deficiency increased body and muscle weights and significantly improved femoral biomechanical strength in *+/oim* mice (Oestreich et al., 2016a). Changes in the physiochemical and microarchitectural properties of bone were also observed in *oim/oim* mice with post-natal pharmacological inhibition of myostatin and other TGF-β ligands with ActRIIB-ligand traps, although to a lesser extent (DiGirolamo et al., 2015; Jeong et al., 2018b). Muscle mass and function were also augmented (DiGirolamo et al., 2015; Jeong et al., 2018a,b).

The *G610C* OI mouse model features a glycine to cysteine substitution, also in the *Col1a2* gene and is phenotypically similar to type I/IV OI patients in an Old Order Amish community in Pennsylvania (Daley et al., 2010). Congenital inhibition of myostatin in heterozygote *+/G610C* mice increased muscle masses beyond Wt levels and improved overall BV (Omosule et al., 2020). Postnatal treatment of *+/G610C* mice with sActRIIB-mFc increased femoral BV and strength, and muscle masses (Jeong et al., 2018a,b).

The *Col1a1^Jrt/+^* OI mouse model is also an autosomal dominant OI mouse model, but differs from the *G610C* and *oim* in that it arose from a splice site mutation in the *Col1a1* gene, leading to an 18 amino acid deletion near the amino terminal end of proα1(I) collagen (Chen et al., 2014). This mouse exhibits skeletal fragility, fragile skin, tendons, and other manifestations clinically akin to combined type III/IV OI (moderate to severe OI) and Ehlers-Danlos Syndrome, (a connective tissue disorder characterized by joint laxity) as seen in a subset of OI/EDS patients (Chen et al., 2014). Treatment with the ACE-2492 ActRIIB ligand trap, designed to bind only myostatin and activin A, dose-dependently increased hindlimb muscle masses (Tauer and Rauch, 2019). A dose-dependent increase in femoral length was also observed with treatment. Additionally, ACE-2492 treated *Col1a1^Jrt/+^* mice exhibited increased periosteal and endocortical femoral diameter; and polar moment of inertia, similar to that seen with ActRIIB-mFc treatment in *+/G610C* mice (Jeong et al., 2018b). However, biomechanical strength was not improved, and no significant changes in vertebrae were observed (Tauer and Rauch, 2019).

The variable responses of these molecularly distinct OI mouse models to potential therapeutic agents was evident in other studies employing therapeutic ligands like TGF-β and sclerostin neutralizing antibodies, thus further necessitating a precision medicine approach to treating OI (Grafe et al., 2016; Kozloff, 2019; Tauer et al., 2019). More importantly, the evidence from these studies further validate myostatin’s potency in muscle regulation. It also suggests that postnatal inhibition of myostatin alone may be inadequate in eliciting significant bone responses in OI. Myostatin inhibition in concert with perhaps other anti-TGF-β ligand therapies, and/or current anabolic and bisphosphonate therapies, may more significantly impact postnatal musculoskeletal properties of OI. Importantly, the study by Oestreich et al. (2016b), suggests that decreasing circulating maternal myostatin levels alters the uterine environment and could potentially induce more skeletal gains in offspring with OI. Thus, further work characterizing the impact of decreasing maternal myostatin levels on fetal musculoskeletal outcomes remains to be undertaken, and a continued awareness that the wide genetic and phenotypic variability that characterizes OI may require multiple treatment approaches to adequately improve associated musculoskeletal deficits.

### Osteoporosis

Significant declines in bone and muscle microarchitecture and function are associated with aging resulting in osteoporosis, osteopenia, and sarcopenia, disorders which constitute a huge health burden on patients and financial liability on the nation (Sànchez-Riera et al., 2010; Blume and Curtis, 2011). Implementing therapeutic strategies to improve musculoskeletal function and health may help mitigate falls and subsequent bone fractures in the elderly.

The data regarding the relationship between serum myostatin and aging in humans is mixed. In a survey of elderly Chinese men and women, plasma myostatin levels correlated with lean body mass (LBM), suggesting that the amount of circulating myostatin is dependent on the amount of lean muscle present (Wu et al., 2018). The research subjects were then divided into a low BMD and high BMD group. The relative abundance of myostatin was lower in the high BMD group compared to the low BMD group, supporting a negative relationship between circulating serum myostatin levels and BMD (Wu et al., 2018). Nonetheless, in anorexia nervosa (AN), an eating disorder characterized by abnormally low body weights, patients will often have low BMD (Pehlivantürk Kızılkan et al., 2018), although with no correlation between their serum myostatin and BMD (Wu et al., 2019). Further, in residents of long term nursing facilities, serum myostatin levels positively correlated with LBM and physical fitness levels (Arrieta et al., 2019). A positive correlation between myostatin levels, greater physical fitness, and higher grip strength was observed in males (Arrieta et al., 2019). Interestingly, improving physical fitness in frail residents increased circulating serum myostatin (Arrieta et al., 2019). In a separate study of community-dwelling individuals 50 years of age and older, frail male participants with low appendicular skeletal muscle mass had higher serum myostatin relative to frail participants with normal skeletal mass (Chew et al., 2019). In addition, a study of 254 older men and women in Japan showed no significant differences in serum myostatin between males and females, although sclerostin and osteocalcin showed sex-specific differences (Moriwaki et al., 2019). In addition, myostatin did not correlate with age in either men or women, nor were any correlations found with height, weight, body mass index (BMI), or grip strength (Moriwaki et al., 2019). The authors cite their inability to distinguish between active and inactive myostatin in their study as a potential reason for the absence of correlations (Moriwaki et al., 2019). These seemingly conflicting studies suggest caution in using serum myostatin as a singular biomarker for a generalized condition like sarcopenia during aging.

Pharmacologic agents are able to alter myostatin serum levels and could potentially impact the homeostatic state of the body (Tsourdi et al., 2019). A clinical trial treating elderly individuals who had experienced recent falls with a monoclonal myostatin antibody decreased circulating myostatin by over 200% in these individuals, and increased appendicular LBM (Becker et al., 2015). Functional assessments also revealed improvements in gait speed and four-step stair climb power (Becker et al., 2015). A trend toward increasing PINP was observed at 24 weeks of treatment. Whole-body BMD was decreased at 36 weeks post-treatment (Becker et al., 2015).

Denosumab and zoledronic acid are potent bone antiresorptive agents that increase BMD and decrease fracture risks in osteoporosis. Denosumab is a human monoclonal antibody against RANKL, and works by inhibiting RANKL-induced osteoclastogenesis (Migliorini et al., 2020); zoledronic acid is a third-generation bisphosphonate that inhibits the key osteoclast enzyme farnesyl pyrophosphate synthetase (Russell et al., 2008; Quesnel et al., 2012); and teriparatide is an osteoanabolic agent also used to improve skeletal integrity. In a study comparing the effect of denosumab on BMD after pre-treatment with either zoledronic acid or teriparatide, Tsourdi et al. (2019) also looked at baseline serum myostatin levels and how they compared after 6 and 12 months of denosumab administration. Teriparatide treatment significantly lowered serum myostatin levels in postmenopausal women. This decrease was maintained 6 and 12 months following Denosumab administration. The administration of denosumab alone in the treatment naïve group of postmenopausal women did not impact serum myostatin levels although lumbar spine BMD was significantly increased (Tsourdi et al., 2019). Additionally, pre-treatment with zoledronic acid did not impact serum myostatin levels at baseline, only in combination with denosumab after 12 months (Tsourdi et al., 2019).

Several murine studies demonstrate an impact of myostatin on bone homeostasis (Arounleut et al., 2013; Chen et al., 2017; Tang et al., 2020). Recently, Tang et al. (2020) reported on the ability of low-intensity pulsed ultrasound (LIPUS) to mitigate bone loss by inhibiting myostatin. LIPUS influences the osteogenic potential of bone and is positively associated with fracture healing (Tang et al., 2020). In this study, ovariectomized (OVX) rats (a model of post-menopausal osteoporosis) exhibited increased serum and muscle myostatin levels relative to sham rats and had lower bone biomechanical and microarchitectural integrity, delayed fracture healing and elevated ActRIIB protein levels. Treatment of OVX rats with LIPUS improved the regenerative capacity of bone and decreased the expression of myostatin, ActRIIB (both mRNA and protein), and the molecules involved in downstream myostatin signaling (Smad2, pSmad2, Smad3, pSmad3), increased bone mechanical strength and promoted fracture healing (Tang et al., 2020). In another study, 12-week recombinant myostatin treatment administered to 12 month old mice decreased trabecular BV, Tb.N, Tb.th, Ct.th, and increased Tb.Sp (Chen et al., 2017). Whereas osteoblast number and bone formation rates were decreased, osteoclast numbers were increased with myostatin administration. Decreased serum bone formation marker PINP (procollagen I N-terminal propeptide) and increased serum bone resorption marker CTX [C-terminal telopeptide of α1(I) collagen] further suggested increased bone loss and decreased bone formation (Chen et al., 2017, p. 8). Lastly, a study of 22 months old mice treated with a myostatin propeptide for 4 weeks revealed muscle hypertrophy without changes in bone microarchitecture, density, and strength (Arounleut et al., 2013).

### Osteoarthritis: Bone in Inflammatory Joint Arthritis

Patients with osteoarthritis have higher serum myostatin, which correlates with disease severity (Zhao et al., 2017). Rheumatoid arthritis patients have substantial myostatin elevation in synovial tissues relative to osteoarthritic patients (Dankbar et al., 2015). Stimulating human synovial cells *in vitro* with the recombinant proinflammatory cytokines rTNFα, interleukin-1 (IL-1), and interleukin-17 (IL-17) increased myostatin expression (Dankbar et al., 2015). Chronic exposure to inflammatory factors in the human TNFα transgene (hTNFtg) mouse also elevated myostatin expression in synovial tissues (Dankbar et al., 2015). Phenotypically, hTNFtg mice develop swollen ankles and eventually loose hindlimb mobility between 9 and 10 weeks of age, symptoms collectively called chronic inflammatory destructive arthritis (Keffer et al., 1991).

To investigate the role of myostatin in inflammation-mediated bone destruction in arthritis, hTNFtg mice with genetic myostatin deficiency (hTNFtg; *Mstn^−/−^*) were generated (Dankbar et al., 2015). A less severe clinical presentation of arthritis was reported in the hybrid hTNFtg; *Mstn^−/−^* mice as compared to hTNFtg mice; evidenced by higher grip strength and delayed paw swelling. Myostatin-deficient arthritic mice also displayed much lower bone erosion (−65%) compared to hTNFtg mice mainly due to decreased osteoclast numbers and inflammation (−45%). In addition, local or intraperitoneal administration of myostatin-specific antibody lessened bone erosion (−58, −31%, respectively), lowered inflammation (−42, −10%, respectively), and reduced osteoclast numbers (−36%) relative to vehicle-treated arthritic mice; suggesting a pharmacological myostatin-specific approach to mitigating inflammation joint destruction may be beneficial in arthritis (Dankbar et al., 2015).

### Muscular Dystrophy

Duchenne muscular dystrophy is an X-linked recessive disorder due to defective or absent dystrophin, a protein critical to myofiber structural integrity and to protecting skeletal muscles from strain-related damage during contraction. Patients with DMD exhibit progressive muscular degeneration, neuromuscular deficits, and poor bone health characterized by increased fracture rates and bone pain (Barzegar et al., 2018). Two mouse models of muscular dystrophy, *mdx* and *dKO*, exhibit considerably lower gene and protein expression of myostatin in the tibialis anterior muscle at both the early disease (8 weeks old) and late disease stages (20–24 weeks) (Zhou et al., 2018). The *mdx* mouse is deficient in dystrophin alone whereas *dKO* is deficient in both dystrophin and utrophin, key proteins in muscular cytoskeletal integrity. As such, both mice have inherent muscle weakness and impaired muscle regeneration (Novotny et al., 2011; van Putten et al., 2020). The *mdx* mouse model is the most commonly used and genetically mimics human DMD although it exhibits a much milder disease phenotype than the more severely affected *dKO* mouse, which has a short lifespan (McDonald et al., 2015; van Putten et al., 2020). Significant detrimental alterations in bone strength, geometry, and microarchitecture are observed in both mouse models although the skeletal phenotype in the *dKO* mouse is much worse (Anderson et al., 1993; Novotny et al., 2011). Additional mouse models of DMD have been generated that more closely phenocopy human DMD (McDonald et al., 2015; van Putten et al., 2020), but current myostatin-related murine DMD data is overwhelmingly *mdx* mouse-dependent.

Inhibiting myostatin and other TGF-β ligands using sActRIIB-Fc in the *mdx* mouse significantly increased body and muscle masses, femoral BV/TV (+80%), Tb.N (+70%), cortical thickness (+14%), volumetric BMD, and femoral biomechanical strength (Puolakkainen et al., 2017). Additional modest improvements in the axial skeleton were also observed in BV, Tb.N, and vBMD along with decreases in trabecular separation in the second lumbar vertebrae (Puolakkainen et al., 2017). Echoing results of studies where myostatin was naturally absent or induced, osteoclast number and the expression of RANKL, a regulator of osteoclast function, were decreased suggesting an inhibitory effect of ActRIIB-Fc on bone resorption (Dankbar et al., 2015; Jeong et al., 2018b; Tauer and Rauch, 2019). The authors further established that expression of the osteoblast and osteocyte markers *Col1a1*, *Opn*, and dentin matrix acidic phosphoprotein 1 (*Dmp-1*) were increased, resulting in enhanced bone formation (Puolakkainen et al., 2017). It is important to note that ActRIIB-Fc traps multiple ligands including myostatin and activin A. Since activin A regulates osteoclasts differentiation, it is possible that the study outcomes reflect the combinatorial effects of inhibiting multiple TGF-β ligands (Gaddy-Kurten et al., 2002; Fowler et al., 2015).

### Diabetes

Myostatin KO mice have reduced fat mass possibly due to increased glucose utilization and increased insulin sensitivity (Guo et al., 2009; Wilkes et al., 2009; Dong et al., 2016). When challenged with a high-fat diet, myostatin propeptide transgenic mice which possess truncated non-functional myostatin proteins, have increased adiponectin, favoring dietary fat utilization for skeletal muscle growth (Zhao et al., 2005; Wilkes et al., 2009). Myostatin deficient type I diabetic mice also have enhanced protein levels of the Glut1 and Glut4 glucose transporters resulting in increased glucose uptake and are protected against type I diabetes mellitus-associated muscle loss (Coleman et al., 2016).

Diabetics have low bone mass, increased risks of bone fractures, and low BMD (Levin et al., 1976; Kalaitzoglou et al., 2016). Diabetic children, in particular, have osteopenic bone with low bone cortical thickness, a likely contributor to the bone loss observed in adulthood (Santiago et al., 1977; McNair et al., 1978). The diabetic bone is characterized by low osteogenic differentiation and decreased bone regeneration (Wallner et al., 2017). In murine diabetic bone, myostatin expression is elevated 9.4-fold, although tibial injury decreased myostatin expression by 34% (Wallner et al., 2017). Local follistatin application to inhibit myostatin at the site of injury enhanced osteoid regeneration and tibial BMD in mice. Moreover, *in vitro*, myostatin inhibition increased proliferation and osteogenesis in mouse adipose-derived stem cells, demonstrated by increased expression of *Alpl*, *Runx2*, and *Pcna in vitro* (Wallner et al., 2017). Further, an 8-week treatment with a polyclonal anti-myostatin antibodies protected diet-induced obese rats from diabetes-associated femoral bone microarchitecture and strength degradation (Tang et al., 2016). Lastly, in a rat model of type 1 diabetes mellitus (T1DM), bone mass, muscle mass, and grip strength were substantially lower relative to healthy controls (Yang et al., 2018). A 6-week weight-bearing exercise regimen improved muscle mass and grip strength, but failed to significantly increase bone mass, although serum levels of the bone resorption marker TRAP, and bone formation marker *Alpl* were lower and higher, respectively (Yang et al., 2018). Hindlimb quadriceps muscles had higher mRNA and protein myostatin levels, which were subsequently lowered with weight-bearing running exercise (Yang et al., 2018). mRNA and protein levels of ActRIIB in femurs were higher in T1DM rats vs. controls, although exercise decreased ActRIIB and Smad2/3 expression (Yang et al., 2018). These data suggest the ability of weight-bearing exercise to decrease both the expression of myostatin and the components of its canonical signaling pathway as a means to improve the diabetic bone phenotype.

A study of 76 patients with type 2 diabetes (T2D) revealed increased myostatin expression in the vastus lateralis muscle, although no differences in serum myostatin were observed (Brandt et al., 2012). Myostatin expression in skeletal muscle was also associated with increased triglycerides, impaired insulin sensitivity, obesity, and poor fitness levels (Brandt et al., 2012). In a separate study of 43 T2D patients, myostatin levels in plasma were much higher and correlated with T2D-associated symptoms: high fasting plasma glucose, serum insulin levels, and BMI (Wang et al., 2012). The pathogenesis of T2D may also include changes in the levels of myostatin in a sex-specific manner given that female T2D patients had higher serum myostatin levels than male patients (Wang et al., 2012).

Conversely, in type 1 diabetes (T1D), no differences in myostatin expression in muscle were observed, although circulating myostatin levels were higher than in controls (Dial et al., 2020). Interestingly, a sex-dependent increase in circulating myostatin was observed, with females exhibiting higher serum myostatin. Circulating myostatin levels correlated with total lean muscle masses, although serum myostatin levels are not considered a pathological response to T1D as the duration of TID and HbA1c levels did not correlate with serum myostatin (Dial et al., 2020).

## Discussion

Functionally and anatomically, muscle and bone are connected. Their shared endocrine functions and pathways are evident in the secretion of autocrine and paracrine ligands (Cianferotti and Brandi, 2014). Further, mechanical forces from muscle dictate bone strength and mass (Frost, 1997, 2003). Thus, during growth, increasing muscle masses stimulate bone modeling with resultant increases in bone strength, whereas disuse and bed rest lead to muscle atrophy and negatively impact bone strength.

The regulatory impact of myostatin in murine muscle mass and fiber number is clearly evidenced by the impact of its inhibition on muscle hypertrophy and regeneration in *mstn^−/−^*, OI, DMD, and osteoporotic mouse models. Pharmacological myostatin inhibition, particularly using ligand traps which inhibit multiple ligands, activin A and myostatin included, more potently effect changes in the musculoskeleton than myostatin inhibition alone, suggesting that a more combinatorial approach of inhibiting specific ActRIIB ligands in addition to myostatin is warranted. Since post-natal myostatin primarily affects muscle fiber hypertrophy, the potency of ligand traps on bone microarchitecture and strength is likely a collective result of increased mechanosensing due to larger muscle loads and changes to bone metabolism including those stimulated by activin A and other TGF-β ligands.

Exciting new reports on the influence of microgravity on muscle and bone have demonstrated that soluble ACVR2B/Fc decoy receptors are capable of improving muscle and bone mass in space, similar to the improvements observed in mice on earth (Lee et al., 2020). Wt mice in the microgravity environment of space (33 days) lost significant amounts of muscle, LBM, and bone mass and microarchitecture relative to Wt mice on earth (Lee et al., 2020; Smith et al., 2020). Grip strength in mice was also significantly decreased in in-flight mice relative to ground control mice (Smith et al., 2020). Remarkably, *mstn^−/−^* mice sent to space had minimal non-significant losses in muscle, but more significant bone loss, although to a lesser degree than Wt mice, suggesting a protective effect of myostatin deficiency even in the microgravity of space. ACVR2B/Fc-treated mice in space had similar vertebral, femoral, and humeral properties as on earth (Lee et al., 2020). Upon return to earth, ACVR2B/Fc treatment fast-tracked recovery of muscle and bone mass in in-flight mice. In another study, the myostatin antibody YN41 protected against loss of muscle mass and grip strength, but failed to protect against microgravity-induced bone loss (Smith et al., 2020). YN41 also did not increase femoral and vertebral BMD in ground control mice (Smith et al., 2020).

When considering bone, differentiation and mineralization in primary murine osteoblasts decreases with increasing concentrations of exogenous myostatin (Hamrick et al., 2007; Elkasrawy et al., 2012; Chen et al., 2017, p. 8). Whereas, recombinant myostatin increases osteoclasts differentiation *in vitro*, resulting in greater numbers of TRAP-positive osteoclasts (Chen et al., 2017, p. 8). Further, although myostatin is known to regulate MSC differentiation (Elkasrawy et al., 2012), and appears to modulate bone cell metabolism *in vitro*, its impact on pre-formed postnatal bone *in vivo* may be minimal (Arounleut et al., 2013), necessitating combinatorial inhibition studies. Nonetheless, not all combinatorial approaches are beneficial. For instance, follistatin, a GDF8/GDF11 inhibitor caused muscle hypertrophy, but weakened bone resulting in fractures in *mstn^−/−^* mice (Suh et al., 2020). Further, in diseases with compromised muscular and skeletal integrity, moderation may be key. The GRippet canine model of combined DMD and myostatin deficiency exhibited worse musculoskeletal outcomes with myostatin treatment (Kornegay et al., 2016). There are also the concerns of exacerbating disease phenotypes in DMD for example by prolonging muscle contractures (St. Andre et al., 2017).

Lastly, for treatments that inhibit myostatin to be pursued in human diseases, beneficial outcomes need to be clearly established. Unfortunately, the data in humans has generated mixed results. In a small study of ambulatory boys with DMD, ACE-031, a fusion protein of ActRIIB and IgG1-Fc only showed a trend toward increasing LBM, BMD and maintenance of the 6-min walk test (6MWT) (Campbell et al., 2017). Another study in 48 healthy postmenopausal women resulted in significant increases in total LBM and thigh muscle volume within a month (Attie et al., 2013). In contrast, several placebo-controlled clinical trials using myostatin inhibitors have failed to provide functional improvements in 6MWT and muscle masses in patients with DMD and inclusion body myositis, thus begging the question: whether improvements to murine musculature with myostatin inhibition translates to humans (Hanna et al., 2019; Polkey et al., 2019; Wagner, 2020). Additional clinical data on patients with muscle atrophy-linked neuromuscular disorders reveal that patients with advanced muscle wasting and atrophy have significant decreases in circulating myostatin levels and myostatin expression in muscle biopsies (Burch et al., 2017; Mariot et al., 2017) in contrast to other studies (Brandt et al., 2012). Myostatin antagonists, employed in clinical trials in DMD, for example, may thus be targeting an already down-regulated ligand, resulting in less significant patient outcomes (Mariot et al., 2017; Rybalka et al., 2020). A recent 2020 review by Rybalka and colleagues highlights this and other biological and therapeutic reasons for the lack of success with using myostatin inhibitors in human DMD (Rybalka et al., 2020).

## Conclusion

**Figure 2:**
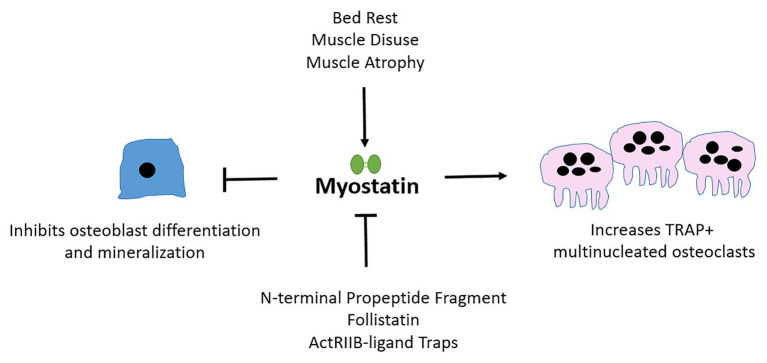
Myostatin affects osteoblast, osteoclast, and osteocyte differentiation and cellular output. Myostatin is secreted by muscle and is upregulated during periods of atrophy or disuse. Myostatin inhibits osteoblast (bone-forming cells) differentiation and activates osteoclast (bone-resorbing) maturation. Follistatin and pharmacological agents that mimic ActRIIB, the receptor through which myostatin signals are examples of natural and artificial inhibitors of myostatin.

To conclude, although exogenous myostatin limits osteoblast differentiation, yet stimulates osteoclast maturation *in vivo* (Figure 2; Table 1); and although the inhibition of myostatin improves muscle and bone properties in murine models of diseases including OI, osteoporosis, and diabetes, the benefits of myostatin therapeutics in human musculoskeletal disorders still need to be validated. First, baseline circulating serum myostatin and muscular myostatin expression levels in respective muscle-related disease pathologies need to be established. Those baseline values can then inform the likelihood that myostatin inhibition therapy has the potential to improve bone and muscle outcomes in humans. Further, it is likely that treatment strategies inhibiting myostatin alongside other TGF-β ligands with musculoskeletal roles, or bisphosphonates (in OI for example), may be more effective.

A current limitation to the studies cited in this review which make comparisons difficult and likely contribute to the mixed treatment outcomes include: variances in antibody formulations, dosage specificity, frequency of administration, treatment durations, the impact of gender, and disease type; all of which affect musculoskeletal outcomes.

## Author Contributions

CO and CP: conceptualization, writing - review and editing, and visualization. CO: writing - original draft. CP: supervision. All authors contributed to the article and approved the submitted version.

### Conflict of Interest

The authors declare that the research was conducted in the absence of any commercial or financial relationships that could be construed as a potential conflict of interest.
